# Role of CBNAAT and ADA in tubercular pleural effusion diagnosis at BMGMC hospital

**DOI:** 10.6026/973206300221098

**Published:** 2026-02-28

**Authors:** Abhishek Tiwari, Darshi Rastogi, Ritu Rani Vinodia, Sachin Parmar

**Affiliations:** 1 Department of Respiratory Medicine, Birsa Munda Government Medical College, Shahdol, Madhya Pradesh, India; 2 Department of Pulmonary Medicine, All India Institute of Medical Sciences, Gorakhpur, Uttar Pradesh, India; 3 Department of Otorhinolaryngology (ENT), Birsa Munda Government Medical College, Shahdol, Madhya Pradesh, India; 4 Department of Community Medicine, V.K.S. Government Medical College, Uttar Pradesh, India

**Keywords:** Tuberculous pleural effusion (TPE), adenosine deaminase (ADA), Cartridge-Based Nucleic Acid Amplification Test (CBNAAT), lymphocytic effusion, diagnostic accuracy

## Abstract

In 2024, 10.8 million new tuberculosis cases were reported globally. Of these, 19% were extrapulmonary (EPTB), meaning the infection occurred outside the lungs. Tuberculous pleural effusion (TPE) is a difficult disease to diagnose because it is only paucibacillary, so consideration should be given to the rapid and easily available diagnostic tools in high tuberculosis prevalence environments. The study was an observational study aimed at assessing diagnostic value of adenosine deaminase (ADA) and Cartridge-Based Nucleic Acid Amplification Test (CBNAAT) in 200 patients with pleural effusion in a tertiary care center. This study established high sensitivity (92.5) but poor specificity (50) of ADA at 40 IU/L cut off; and low sensitivity (28.35) but high specificity (50) of CBNAAT at 40 IU/L cut off. Effusions of tuberculosis were 67 percent with males being the most at 68 percent in the age group of 41 to 50. There was a strong positive correlation between the lymphocyte predominance with the ADA levels (r=0.71, P=0.003) and the CBNAAT positivity (r=0.95, P=0.0015). Thus, we show that ADA is still a useful screening method in tuberculous pleural effusion, especially when it is used in conjunction with lymphocyte-predominant exudates and CBNAAT is a confirmatory study with the further benefit of identifying rifampicin resistance.

## Background:

Tuberculosis (TB) is an important worldwide health challenge as 10.8 million new cases of tuberculosis were identified in the world in 2024, of which 19% was extrapulmonary tuberculosis (EPTB) [1]. Tuberculous pleural effusion (TPE) is an expression of EPTB that is widely observed especially in the developing world where it is the major etiology of pleural effusions [2]. In the high TB burden nations, TPE is associated with 23.5% to 82.4% of the pleural effusions diagnosed using medical thoracoscopy, with India having 23.5% and South Africa having 82.4% [3]. Characteristic features of the disease are the acute development of fever, cough and pleuritic chest pain and the pleural fluid is usually dominated by lymphocytes [4]. TPE is a challenging diagnosis because of a pauci-bacillary nature of the disease that results in low detectability using conventional diagnostic strategies [5]. Diagnosis is based on the gold standard, which is the identification of Mycobacterium tuberculosis in pleural fluid or pleural tissue, or histological evidence of caseating granulomata in pleural biopsy, preferably with the presence of acid-fast bacilli [6]. Nevertheless, acid-fast bacilli smear microscopy has a sensitivity of 0-5% in pleural fluid whereas mycobacterial culture, although more sensitive (11-58%), has a turnaround time of 4-8 weeks [7]. Moreover, the diagnostic challenges are also complicated by the lack of access to and the skills to perform, pleural biopsies using closed needle biopsy, thoracoscopy or open surgery surgeries, especially in resource-restricted environments [8]. The TPE has been identified as a valuable biomarker that can be used to diagnose the condition as adenosine deaminase (ADA) is an enzyme in purine metabolism and was mostly produced by activated T lymphocytes [9]. Different meta-analyses have shown pleural fluid ADA has excellent diagnostic capacity of TPE, with sensitivities of 89 to 95.5% and specificities of 92 to 93.4% with a cut point of 40 U/L [10]. Palma *et al.* did a meta-analysis of 16 studies with 4,147 patients and reported that ADA TPE has a sensitivity of 93% and 92% specificity [11]. Nonetheless, ADA can also be high in empyema, parapneumonic effusion and some malignancies, especially lymphoma, which can restrict its specificity in some clinical situations [9].

Cartridge-Based Nucleic Acid Amplification Test (CBNAAT), also called the Xpert MTB/RIF assay, is an important development in the fast and molecular diagnosis of tuberculosis [12]. Although the sensitivity (98%) and specificity (98%) of CBNAAT are excellent in smear-positive pulmonary tuberculosis, its capability in TPE is less predictable [13]. Another systematic review and meta-analysis with pleural fluid samples by Sehgal *et al.* has given pooled sensitivities of 51.4% and 22.7% with culture and composite reference standard respectively, although specificity remained high at 98.6% and 99.8% respectively [12]. The reduced sensitivity in pleural fluid is explained by the fact that TPE is pauci-bacillary with loads of bacilli at times below the detection limit of the test [14]. The recent reports have discussed the approach to enhance CBNAAT diagnostic achievement in TPE such as concentrated sample of pleural fluid and fresh specimen, which have already demonstrated significantly greater sensitivities [15]. A newer Xpert MTB/RIF Ultra assay test has been shown to have a high diagnostic accuracy as compared to the initial Xpert assay with a meta-analysis by Aggarwal *et al.* showing the sensitivities of 68 percent and the specificities of 97 percent when mycobacterial culture is used as the reference [9]. CBNAAT has moderate sensitivity but high specificity (>95%) and thus is a good rule-in test (when positive) and has the ability to detect rifampicin resistance in hours, which is a significant positive attribute when compared to traditional culture-based drug susceptibility testing. Moreover, the CBNAAT-positive findings are associated with the high level of ADA (>40 U/L) and lymphocyte-dominant effusions, which are in line with the pathogenesis of TPE [16, 17]. This study advances current knowledge by evaluating the synergistic diagnostic performance of combined CBNAAT and ADA testing. Therefore, it is of interest to evaluate the diagnostic significance of CBNAAT and ADA in patients with tuberculous pleural effusion, assessing their individual and combined performance in a tertiary care setting.

## Materials and Methods:

## Study design:

It was an observational study done at the Department of Respiratory Medicine, BMGMC Hospital, Shahdol, in the period between January 2022 and June 2025. The study included 200 patients who came with symptoms, medical history and radiologic evidence indicating that they had pleural effusion. This period was characterized by the admission of patients in the hospital, or in the outpatient department (OPD).

## Inclusion criteria:

The study included patients who were diagnosed with pleural effusion and were proved by using the following methods: Clinical examination, Chest X-ray and Ultrasonography Indications Diagnostic thoracocentesis to analyze the fluid with an exudative nature, according to Light.

## Exclusion criteria:

The following were used as the exclusion criteria used in the study of patients: Patients who failed to offer consent to thoracocentesis, Transudative effusion of the pleura and patients with hemothorax (bleeding pleural effusion).

## Methodology:

A total of 200 patients diagnosed with pleural effusion were enrolled in the study after obtaining informed consent. Each patient underwent a detailed medical history evaluation and a comprehensive clinical examination. Sputum samples were collected and analyzed for acid fast bacilli (AFB) using the smear method and cartridge based nucleic acid amplification test (CBNAAT).Diagnostic thoracocentesis was performed under strict aseptic precautions. The aspirated pleural fluid was subjected to biochemical analysis, including estimation of glucose, protein, and lactate dehydrogenase (LDH) levels. Microbiological evaluation was carried out with Ziehl-Neelsen staining for AFB, and cytological assessment included measurement of total and differential leukocyte counts. Corresponding serum protein and serum LDH levels were determined for comparative assessment. All patients who fulfilled Light's criteria for exudative pleural effusion were further evaluated for adenosine deaminase (ADA) activity and CBNAAT results.

Diagnosis of Tuberculous Pleural Effusion Tuberculous pleural effusion was diagnosed based on one or more of the following criteria:

[1] Detection of Mycobacterium tuberculosis in pleural fluid or sputum by Ziehl-Neelsen staining or CBNAAT.

[2] Clinical presentation consistent with tuberculosis, with exclusion of other potential causes.

[3] ADA activity greater than 40 IU/L in exudative, lymphocytic pleural effusion (as per Light's criteria).

[4] Definite clinical and radiological improvement within two months of exclusive anti tubercular therapy (ATT).

## ADA measurement:

Pleural fluid ADA activity was determined by the conventional method. A cut-off of ADA value was set at 40 IU/L to diagnose tuberculous pleural effusion.

## Results and Discussion:

The sample size used was 200 patients with pleural effusion, with a male to female ratio of 2.125:1 (136 males and 64 females, (Table 1). The average age was 41.57 years (minimum: 2 years to maximum age 85 years) and tubercular effusions were most common in the 41-50 year age bracket (26.5%). The most frequent presenting symptoms were cough (91) and fever (89) and shortness of breath (82) and chest pain (81) (Table 2). The effusions were mostly right-sided (78%) and 82 percent of the patients were low-literacy level smokers. Etiological examination showed that tubercular effusions were found in 67 percent, parapneumonic effusions in 23.5 percent, empyema in 5 percent and malignant effusion in 4.5 percent (Table 5). Lymphocyte pre-eminence (>50) was in 67 percent of effusions. With a cut-off of ADA of 40 IU/L, 194 cases (97% ADA>40) of which 185 were tubercular of which 9 were malignant gave a sensitivity of 92.5 and specificity of 50% (Table 3 and Table 4). Pleural fluid CBNAAT was positive in 58 cases (28.35% sensitivity, 50% specificity), with 3 cases of ADA under 40 IU/L; all of them were rifampicin-sensitive (Table 4). Sputum AFB positivity was 6%. A positive significant relationship was identified between the number of lymphocytes and the two ADA results (r=0.71, P=0.003) and CBNAAT positivity (r=0.95, P=0.0015) (Table 6).

There was significant positive correlation seen with ADA detected patients and lymphocyte count, r=0.71, P-value=.003HS (P<0.05). Significant positive correlation seen with CBNAAT detected patients and lymphocyte count, r=0.95, P-value=0.0015 HS (P0.05). This observational study aimed to evaluate the diagnostic utility of ADA and CBNAAT in patients with pleural effusion, focusing particularly on tuberculous effusions. The study included 200 patients, predominantly male (136 males and 64 females), with an average age of 41.57 years. The highest prevalence of tubercular effusions was observed in the 41-50 year age group (26.5%). The most common symptoms were cough (91%), fever (89%), shortness of breath (82%) and chest pain (81%). Right-sided effusions were more common (78%) and 82% of the patients were smokers with low literacy levels. Etiological analysis revealed that 67% of the effusions were tubercular, followed by parapneumonic effusions (23.5%), empyema (5%) and malignant effusion (4.5%).The male predominance (68%) in the present study is consistent with published literature, where male-to-female ratios of 2:1 are commonly reported in tuberculous pleural effusions. Zhai *et al.* [2] documented similar male predominance with a ratio of 2:1 in their comprehensive review. The mean age of 41.57 years in the current study aligns with findings from high tuberculosis burden areas, where TPE predominantly affects younger individuals, with mean ages ranging from 34-49 years. Das *et al.* [18] demonstrated that tuberculous effusions occur predominantly in patients less than 40 years (76.6%), in stark contrast to malignant effusions which affect older individuals. The high prevalence of smoking (82%) observed in this study is supported by Tewatia *et al.* [19] who established a significant association between tobacco smoking and TPE, with odds ratios of 19.22 for cigarette smoking and 4.57 for combined cigarette or beedi smoking. The right-sided predominance (78%) observed exceeds previously published rates of 55% by Vorster *et al.* [20]. The diagnostic performance of ADA was particularly notable, with 97% of the patients showing ADA levels greater than 40 IU/L, which resulted in a sensitivity of 92.5% for diagnosing tuberculous effusions. However, the specificity was only 50%, indicating that while ADA is a highly sensitive test; its low specificity limits its ability to conclusively differentiate between tuberculous and other causes of exudative effusion. The sensitivity observed in this study is comparable to the meta-analysis by Liang *et al.* [21] which included 63 studies and reported a pooled sensitivity of 92% (95% CI: 0.90-0.93) and specificity of 90% (95% CI: 0.89-0.91) for ADA in diagnosing tuberculous pleurisy.

Similarly, Castro *et al.* [22] conducted a meta-analysis of Spanish population studies, demonstrating ADA sensitivity of 93% and specificity of 92%. The lower specificity in the current study (50%) contrasts with these meta-analyses and may reflect differences in the prevalence of conditions that can elevate ADA levels, such as empyema and certain malignancies. Rosa *et al.* [23] reported sensitivity and specificity of 92.3% and 97.3% respectively at ADA cutoff of 40 IU/L in their prospective study. The findings support the utility of ADA in screening for tuberculous pleural effusion, though further confirmation is needed, particularly due to its moderate specificity in this cohort. On the other hand, CBNAAT had a much lower sensitivity of 28.35%, despite the fact that it had 50% specificity. This lower sensitivity suggests that CBNAAT may not be as reliable in pleural effusion samples as it is in sputum, possibly due to a lower bacterial load in pleural fluid or issues with sample collection. The CBNAAT positivity rate of 29% observed in this study aligns closely with reported literature values ranging from 18.5% to 45.3% Nishal *et al.* [15] documented CBNAAT sensitivity of 32.3% and specificity of 100% against a composite reference standard in extrapulmonary tuberculosis, underscoring the paucibacillary nature of extrapulmonary specimens. The limited sensitivity in pleural fluid samples reflects the inherently low bacterial load in tuberculous pleurisy, a finding consistently reported in prior study done by Kumar *et al.* [24]. Notably, three cases with ADA levels below 40 IU/L were rifampicin-sensitive, highlighting the need to combine ADA testing with microbiological confirmation for optimal diagnostic accuracy. A strong positive correlation emerged between pleural fluid lymphocyte count and both ADA levels (r=0.71, P=0.003) and CBNAAT positivity (r=0.95, P=0.0015), positioning lymphocytic predominance as a robust tuberculosis indicator (Kumar *et al.*). Koh *et al.* [25] similarly reported an inverse association between lymphocyte percentage and positive effusion culture (adjusted OR 0.93, P=0.001), affirming the characteristic lymphocytic pattern in tuberculous pleurisy. These observations emphasize integrating cytological, biochemical, and microbiological assessments for comprehensive pleural effusion evaluation. Despite ADA's high sensitivity, limitations persist, including its modest specificity and CBNAAT's constrained pleural fluid sensitivity also observed by Kumar *et al.* [24]. The single-center design further limits generalizability. Multicenter studies with larger cohorts are warranted to refine diagnostic algorithms for tuberculous pleural effusion.

## Conclusion:

In endemic regions, TPE is primarily a disease of young adults and smokers. ADA serves as an excellent screening tool due to its high sensitivity, while CBNAAT provides definitive microbiological confirmation and drug resistance data. The strong correlation between lymphocyte density and these markers reinforces cytological analysis as a cornerstone of TPE diagnosis.

## Figures and Tables

**Table 1:** Age wise distribution of study population

**Age Group(in years)**	**Males**	**Females**	**Percentage**
10-Jan	3	1	2%
20-Nov	14	12	13%
21-30	29	9	19%
31-40	18	8	13%
41-50	20	13	26.50%
51-60	21	10	15.50%
61-70	20	9	14.50%
71-80	9	2	5.50%
81-90	2	0	1%
Total	136	64	100%

**Table 2:** Clinical features in study population

**Symptoms**	**No. of Cases**	**Percent**
Cough	182	91%
Fever	178	89%
SOB	164	82%
Chest Pain	162	81%
Weight Loss	100	50%
Loss of Appetite	96	48%

**Table 3:** ADA level in pleural fluid

**Pleural Fluid ADA(IU/L)**	**No. of Cases**	**Percent**
<=40	6	3%
>40	194	97%
Total	200	100%

**Table 4:** ADA and CBNAAT in study

**ADA(IU/L)**	**No. Of Cases**	**CBNAAT MTB Detected**	**CBNAAT MTB not Detected**
ADA>40	194	55	139
ADA<40	6	3	3
TOTAL	200	58	142

**Table 5:** Distribution of patients according to etiological diagnosis

**Etiological**	**NO. of Patients**	**Percent**
Lymphocytic Effusion	134	67%
Suggestive of Tuberculosis		
Malignant Effusion	9	4.50%
Empyema	10	5%
Parapneumonic Effusion	47	23.50%
Total	200	100%

**Table 6:** Pleural fluid lymphocyte wise ADA and CBNNAT in study population

**Lymphocyte**	**Cases**	**ADA**		**CBNAAT**	
		>40	<40	MTB Detected	MTB not Detected
50-59	2	1	1	1	1
60-69	12	11	1	4	8
70-79	16	15	1	6	10
80-89	32	31	1	11	21
90-100	92	90	2	26	66
